# Retraction: A study on the luminescence properties of gamma-ray-irradiated white light emitting Ca_2_Al_2_SiO_7_:Dy^3+^ phosphors fabricated using a combustion-assisted method

**DOI:** 10.1039/d2ra90104h

**Published:** 2022-10-19

**Authors:** Geetanjali Tiwari, Nameeta Brahme, Ravi Sharma, D. P. Bisen, Sanjay K. Sao, S. J. Dhoble

**Affiliations:** School of Studies in Physics and Astrophysics, Pt. Ravishankar Shukla University Raipur C.G. India geetanjali.tiwari10@gmail.com namitabrahme@gmail.com; Department of Physics, Govt. Arts and Commerce Girls College Devendra Nagar Raipur C.G. India; Department of Physics, RTM University Nagpur Maharashtra India

## Abstract

Retraction of ‘A study on the luminescence properties of gamma-ray-irradiated white light emitting Ca_2_Al_2_SiO_7_:Dy^3+^ phosphors fabricated using a combustion-assisted method’ by Geetanjali Tiwari *et al.*, *RSC Adv.*, 2016, **6**, 49317–49327, https://doi.org/10.1039/C6RA04913C.

The Royal Society of Chemistry hereby wholly retracts this *RSC Advances* article due to concerns with the reliability of the data.

Following the previous publication of a correction, which was published after an institutional investigation, to correct errors in Fig. 10(a, b), 11(a, b), 12(a, b), 15, 18 and 19, further instances of data concerns have been identified within the corrected Fig. 10a, 11a and 12a. The corrected Fig. 10a, 11a, and 12a contain sections of identical noise and these concerns undermine the integrity of this article.

Given the significance of these concerns, the findings presented in this paper are no longer reliable.

This retraction supersedes the information provided in the correction related to this article and the correction is no longer valid.

The authors were informed about the retraction of the article. Nameeta Brahme has not agreed with the decision, the other authors have not responded.

Signed: Laura Fisher, Executive Editor, *RSC Advances*

Date: 13/10/2022

## Supplementary Material

